# In situ diffuse large B‐cell lymphoma in haemorrhoidectomy tissue

**DOI:** 10.1002/jha2.521

**Published:** 2022-06-22

**Authors:** Osamu Nakai, Fumihiko Kono, Takashi Miyoshi, Shinsaku Imashuku

**Affiliations:** ^1^ Department of Surgery Uji‐Tokushukai Medical Center Uji Kyoto Japan; ^2^ Division of Pathology Uji‐Tokushukai Medical Center Uji Kyoto Japan; ^3^ Division of Hematology Uji‐Tokushukai Medical Center Uji Kyoto Japan; ^4^ Department of Laboratory Medicine Uji‐Tokushukai Medical Center Uji Kyoto Japan

**Keywords:** diffuse large B‐cell lymphoma, haemorrhoid, haemorrhoidectomy, in situ lymphoma

1

A 35‐year‐old Japanese female (gravida 2, para 2), who was negative for herpes simplex virus, hepatitis C virus, human immunodeficiency virus, and human T‐cell Leukemia Virus Type 1, had a 4‐month history of prolapsed haemorrhoids, which began after the birth of her first child (Figure 1A). She received two haemorrhoidectomies. The first, on August 2017, revealed that tissue in the 6 o'clock direction was nonmalignant; however, a second (conducted in November 2018) revealed that the tissue in the 12 o'clock direction showed diffuse large B‐cell lymphoma (DLBCL; Figure 1B–F), which had the following immunophenotype: CD3−, CD5−, CD10−, CD20+, CD79a+, BCL‐2−, BCL‐6−, MUM1+, and Ki‐67 (70% positive), which is consistent with a nongerminal center B‐cell type. Unfortunately, no extra tissues were available for further karyotype or molecular studies.

**FIGURE 1:**
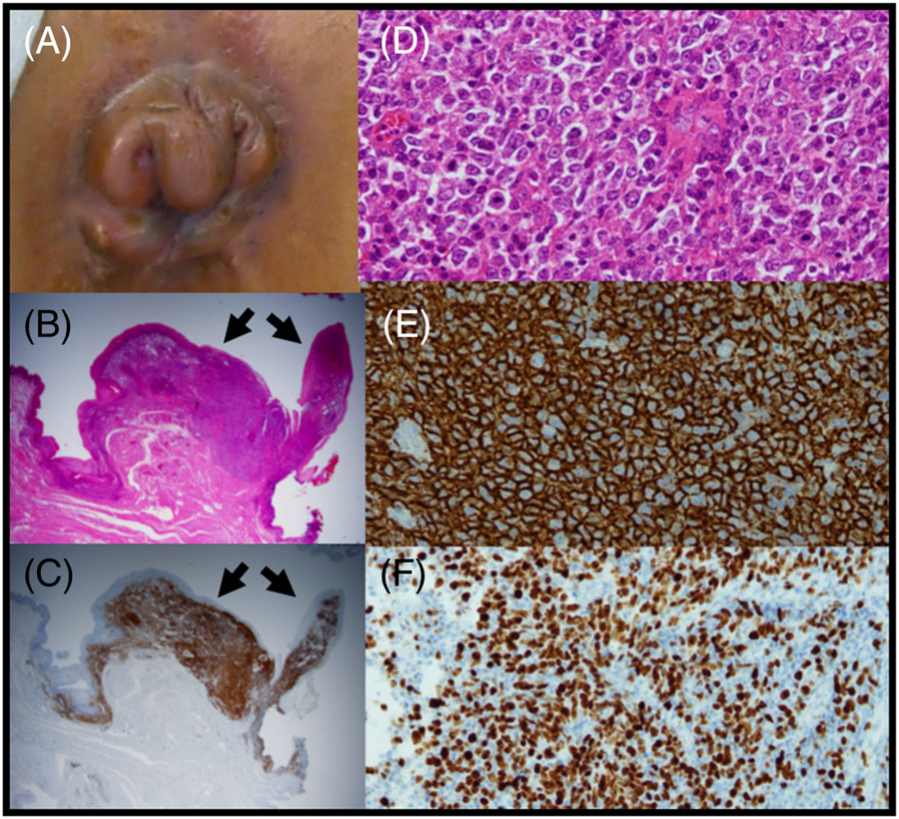
Photo of the haemorrhoids (A). Histology of the haemorrhoidectomy tissue under a low‐power field microscope (B; H&E staining; arrows indicate the lymphoma areas. Original magnification ×1.25) and (C; CD20 staining; arrows indicate lymphoma areas. Original magnification ×1.25). Sizes of lymphoma areas were measured as 5 mm × 2 mm and 5 mm × 1 mm, respectively. Histology of DLBCL under a high‐ power field microscope (D; H&E staining. Original magnification ×400), (E; CD20 staining. Original magnification ×400); and (F; Ki‐67 staining. Original magnification ×200)

The patient had no constitutional symptoms, palpable cervical/inguinal lymph nodes, or hepatosplenomegaly. Laboratory tests revealed the following: normal blood counts (white blood cell count, 7.5×10^9^/L; haemoglobin, 134 g/L; platelet count. 240×10^9^/L), no inflammatory signs (serum C‐reactive protein, 7 mg/L), normal serum levels of soluble interleukin‐2 receptor (0.182×10^6^ U/L; reference, 0.220–0.530), and normal serum immunoglobulin levels (IgG, 11.27×10^3^ mg/L; IgA, 3.49×10^3^ mg/L; IgM, 1.05×10^3^ mg/L). Her hepatic and renal functions were normal. ^18^F‐fluorodeoxyglucose‐positron emission tomography/computed tomography (PET/CT) revealed no additional pathologic lesions. The patient was diagnosed with in situ DLBCL and was put under surveillance. Three and a half years later, she is doing well without recurrence.

The discovery of lymphoma in a haemorrhoid tissue was an incidental diagnosis in this case. Although gastrointestinal lymphoma is common, it is a rare malignancy in the anal margin and perianal skin. Only a few reports describe malignant lymphoma developing in external or internal haemorrhoids [1, 2]. One case was a 33‐year‐old female who received haemorrhoidectomy which was diagnosed with B‐cell lymphoma. In this case, an additional lesion was found when abdominal perineal rectal amputation was performed one month later. This patient was still alive at the time of the report [1]. The other case was a 64‐year‐old man with a history of haemorrhoidal diseases, who developed a perianal mass mimicking an external thrombosed haemorrhoid in association with conglomerate inguinal lymph nodes, both tissues were diagnosed as mantle cell lymphoma [2]. This patient died of comorbidities after surgery [2].

In summary, malignant lymphoma in haemorrhoids is extremely rare. However, if we had not tested the histopathology of haemorrhoidectomy tissue, DLBCL might be overlooked. Luckily in our case, the DLBCL was diagnosed at the early phase as in situ neoplasm limited within a haemorrhoid and the patient survived without treatment. Though, in the past, routine histopathologic evaluation of haemorrhoids was thought unnecessary, because the incidence of histologic abnormalities (other than the expected lesions) is very low (13/914 (1.4%)) [3], our experience clearly necessitates histological examination of haemorrhoidectomy specimens. Whenever malignant tissue is identified in haemorrhoidectomy specimens, detailed examinations are urgently required to search if the malignancy is extended beyond hemorrhoids.

## FUNDING INFORMATION

The authors received no specific funding for this work.

## ETHICS STATEMENT

The work described in this study was carried out in accordance with the Declaration of Helsinki.

## PATIENT CONSENT STATEMENT

Written informed consent to publish was obtained from the patients.

## CONFLICT OF INTEREST

The authors declare they have no conflicts of interest.
